# Supplementary material for: Effect of Peony (Paeonia ostii) Seed Meal Supplement on Enzyme Activities and Flavor Compounds of Chinese Traditional Soybean Paste during Fermentation
Source: Foods. 2023 Aug 24;12(17):3184. doi: 10.3390/foods12173184 (PMC10486673; doi:10.3390/foods12173184)
Supplement: Supplementary file 1 [file foods-12-03184-s001.zip › foods-2507084-supplementary.pdf]

Table S1. The quantitative and qualitative results of volatile flavor compounds in SH and PH samples

CAS number	Flavor compound	Oodor threshold (μg/kg)[1]	Aroma descriptors[2]	Concentration(μg/kg)		OAVs	
				SH	PH	SH	PH
	Aldehydes						
124-19-6	Nonanal	1.1	rose, orange	5.20±0.93	-[3]	4.728654	-
590-86-3	3-Methylbutanal	1.1	chocolate, fatty	-	266.08±49.37	-	241.888330
597-31-9	3-Hydroxy-2,2-dimethylpropanal	-	-	0.75±0.10	-	-	-
66-25-1	Hexanal	5	grass, leafy	2.16±0.09	9.12±1.07	6.758144	1.824060
98-01-1	Furfural	770	baked bread	-	4.29±1.39	-	0.005567
Subtotal				8.11	279.49	-	-
	Ketones						
123-19-3	4-Heptanone	8.2	cheese	-	18.31±3.52	-	2.233508
15726-15-5	3-Methyl-4-heptanone	0.05	-	-	7.88±0.20	-	157.764343
19550-10-8	3,4-Dimethyl-2-hexanone	-	-	-	2.95±1.24	-	-
5857-36-3	2,2,4-Trimethyl-3-Pentanone	-	-	-	4.04±1.95	-	-
2918-13-0	1-Hepten-3-one	0.04	-	0.92±0.06	-	23.053997	-
Subtotal				0.92	33.18		
	Alcohols						
104-76-7	2-Ethyl-1-hexanol	198	-	1.41±0.22	7.30±1.18	-	0.036869
108-93-0	Cyclohexanol	160000	-	0.44±0.08	-	0.000003	-
111-27-3	1-Hexanol	391	sweet,alcoolic	0.31±0.05	-	0.000804	-
111-70-6	1-Heptanol	5.4	leafy, herbal	20.40±0.08	-	3.777015	-
111-87-5	1-Octanol	100	rose, mushroom	3.77±0.91	6.03±1.25	0.060311	0.089646
112-42-5	1-Undecanol	86	-	-	68.18±4.58	-	0.792767

123-51-3	3-Methyl-1-Butanol	4	whiskey, banana	23.34±1.63	75.07±7.44	3.826459	18.768048
13231-81-7	3-Methyl-1-hexanol	-	-	0.35±0.09	-	-	-
18409-17-1	2-Octen-1-ol	100	cirrus, vegetable	1.14±0.07	-	0.011415	-
24070-77-7	2-Methylcyclopentanol	-	-	-	5.13±1.02	-	-
2919-23-5	Cyclobutanol	4600	-	-	4.38±2.40	-	0.000952
3391-86-4	1-Octen-3-ol	1.5	mushroom	18.09±1.95	-	12.061223	-
35854-86-5	(*Z*)-6-Nonen-1-ol	1	honeysuckle	0.47±0.09	-	0.470918	-
4435-50-1	1,2,3-Butanetriol	-	-	-	6.66±0.94	-	-
4798-44-1	1-Hexen-3-ol	500	vinegar	-	23.17±4.03	-	0.046331
50639-00-4	2-Ethylhex-2-enol	-	-	-	6.84±0.52	-	-
584-02-1	3-Pentanol	4125	herbal	1.26±0.21	-	0.000306	-
918-85-4	3-Methyl-1-penten-3-ol	-	-	-	2.08±1.28	-	-
Subtotal				70.99	204.84		
	Acids						
1070-83-3	3,3-Dimethylbutyric acid	920	-	-	2.82±0.09	-	0.003060
142-62-1	Hexanoic acid	35.6	sour, cheese	0.36±0.06	-	0.009972	-
149-57-5	2-Ethylhexanoic acid	27000	-	27.32±2.76	41.31±15.03	0.001012	0.001530
334-48-5	Decanoic acid	130	fatty	0.54±0.12	-	0.004150	-
503-74-2	3-Methylbutanoic acid	12	cheese	117.90±21.21	38.53±0.07	9.825023	3.211118
541-47-9	3-Methyl-2-butenoic acid	14000	-	0.64±0.08	-	0.000046	-
57-10-3	Hexadecanoic acid	1100	waxy	43.71±2.71	216.55±37.30	0.004371	0.196868
64-19-7	Acetic acid	180000	vinegar	11.70±9.23	44.01±4.01	0.000065	0.000245
79-31-2	2-Methylpropanoic acid	29000	cheese, buttery	8.06±1.94	1.66±0.50	0.000278	0.000057
Subtotal				210.23	408.93		
	Esters						

108-05-4	Ethenyl acetate	1400	-	-	6.60±0.72	-	0.004715
108-64-5	Ethyl 3-Methylbutanoate	0.1	apple, pineapple	2.64±0.23	8.24±1.66	24.016361	82.436851
110-38-3	Ethyl decanoate	5	apple, brandy	0.70±0.11	-	0.140680	-
111-11-5	Methyl octanoate	200	orange, herbal	0.29±0.09	-	0.001452	-
111-82-0	Methyl dodecanoate	1.5	-	0.44±0.04	-	-	0.291022
112-39-0	Methyl hexadecanoate	2000	waxy	11.17±3.52	7.49±0.48	0.005585	0.003746
112-63-0	Methyl linoleate	-	fatty, woody	11.38±4.22	-	-	-
123-92-2	Isopentyl acetate	0.15	fruity	-	18.90±6.10	-	125.991063
1731-84-6	Methyl nonanoate	40	pear, wine	-	10.36±3.24	-	0.258899
1937-62-8	Elaidic acid methyl ester	-	-	3.83±0.39	-	-	-
2308-38-5	*tert*-Butyl butanoate	-	-	-	2.79±0.77	-	-
2432-77-1	Methyl thiohexanoate	0.3	nutty, buttermilk	-	6.70±0.47	-	22.325428
2438-20-2	2-Methyl butyl propionate	28	-	-	2.47±0.55	-	0.088193
2463-02-7	Methyl11,14-eicosadienoate	-	-	-	7.41±2.32	-	-
27625-35-0	Isopently 2-methylbutanoate	8.6	blueberry, apple	2.06±0.10	-	0.239632	-
301-00-8	Methyl linolenate	-	-	0.53±0.12	3.28±0.15	-	-
3050-69-9	Vinyl hexanoate	-	-	0.95±0.23	-	-	-
539-82-2	Ethyl pentanoate	0.3	apple, pineapple	0.66±0.13	-	2.215631	-
659-70-1	Isopentyl 3-methylbutanoate	20	apple,fruity	1.54±0.63	-	0.077121	-
7452-79-1	Ethyl 2-methylbutyrate	0.06	green, fruity	1.21±0.13	-	20.234830	-
Subtotal				37.41	74.24		
	Furans						
1192-62-7	2-Acetylfuran	10000	almond, cocoa	-	6.90±1.03	-	0.000690
1927-59-9	2-*tert*-Butoxytetrahydrofuran	-	-	0.49±0.20	-	-	-

3208-16-0	2-Ethyfuran	8000	coffee, nutty	-	7.84±0.52	-	0.000980
3777-69-3	2-Pentylfuran	5.8	fruity,beany	2.61±0.17	48.38±0.35	0.449631	8.341719
534-22-5	2-Methylfuran	200	chocolate	-	22.04±2.15	-	0.110196
Subtotal				3.1	85.16		
	Pyrazines						
108-50-9	2,6-Dimethylpyrazine	400	nutty, coffee	5.51±1.08	3.45±1.69	0.013787	0.008621
123-32-0	2,5-Dimethylpyrazine	80	roasted nuts	9.90±6.73	-	0.123694	-
13925-03-6	2-Ethyl-6-methylpyrazine	40	camphor, menthol	1.84±0.30	-	0.045947	-
13925-09-2	2-Methyl-6-vinylpyrazine	-	hazelnut	6.37±1.92	35.72±1.85	-	-
14667-55-1	Trimethylpyrazine	23	nut, cocoa	1.74±0.40	5.51±0.84	0.075491	0.239441
Subtotal				25.36	44.68		
	Alkanes						
15869-96-2	4,5-Dimethyloctane	-	-	-	4.84±0.27	-	-
16747-31-2	3,3,4-Trimethylhexane	-	-	-	3.73±1.25	-	-
17302-32-8	Nonane,3,7-dimethyl-	-	-	-	32.06±8.86	-	-
5076-19-7	2,3-Epoxy-2-methylbutane	-	-	-	2.29±0.72	-	-
61141-72-8	Dodecane,4,6-dimethyl	-	-	-	23.04±0.63	-	-
629-62-9	Pentadecane	13000000	waxy	-	65.08±7.38	-	0.000005
7154-80-5	3,3,5-Trimethylheptane	-	-	-	4.21±1.52	-	-
7335-17-3	3,5-Dimethyl-4-octanone	-	-	-	8.88±0.93	-	-
75-83-2	2,2-Dimethylbutane	70000	-	1.01±0.27	2.42±0.32	0.000014	0.000035
Subtotal				1.01	146.55		
	Olefin						
4316-65-8	3,5,5-Trimethyl-1-hexene	-	hazelnut	-	14.02±0.27	-	-

Subtotal					14.02		
	Aromatic compounds						
100-51-6	Benzyl alcohol	2546.21	rose	-	9.85±0.89	-	0.003868
60-12-8	2-Phenylethanol	390	rose	15.03±1.91	41.99±0.91	0.107664	0.038538
101-41-7	Methyl phenylacetate	-	floral, almond	6.39±0.69	-	-	-
103-65-1	Propylbenzene	177.12	-	3.53±1.15	24.23±0.12	0.019903	0.136826
103-82-2	Phenylacetic acid	12000	-	1.19±0.27	-	0.000099	-
106-42-3	1,4-Dimethylbenzene	530	-	4.07±0.72	20.47±13.53	0.007677	0.038630
108-38-3	1,3-Dimethylbenzene	1000	-	14.91±0.67	29.34±1.70	0.014906	0.029341
108-67-8	1,3,5-Trimethylbenzene	3	-	6.01±0.03	-	2.001758	-
1138-52-9	3,5-Bis(*tert*-butyl)phenol	-	-	0.48±0.16	-	-	-
118-69-4	2,6-Dichlorotoluene	-	-		32.21±16.49	-	-
118-93-3	1'-Hydroxyacetophenone	-	hawthorn, herbal		11.81±3.52	-	-
122-78-1	2-Phenylethanal	6.3	hyacinth, clover	30.84±0.43	194.66±22.95	4.895550	30.897774
13679-41-9	3-Phenylfuran	-	-	0.49±0.14	-	-	-
19398-61-9	2,5-Dichlorotoluene	-	-	85.61±14.08	437.23±130.96	-	-
2077-46-5	2,3,6-Trichlorotoluene	-	-	-	18.23±1.06	-	-
2142-73-6	2',5'-Dimethylacetophenone	-	-	-	97.82±0.83	-	-
21834-92-4	Cocoa hexenal	-	cocoa	-	38.06±3.28	-	-
25552-17-4	1-Phenyl-5-methyl-1-hexanone	-	-	-	2.98±1.09	-	-
28715-26-6	4,7-dimethylbenzofuran	-	hawthorn	2.13±0.88	401.98±51.49	-	-
32768-54-0	2,3-Dichlorotoluene	-	-	156.51±26.68	1611.20±108.03	-	-
4411-89-6	2-Phenyl-2-2butenal	-	beany, honey	-	8.51±1.91	-	-
526-73-8	1,2,3-trimethyl-Benzene	-	-	6.61±1.52	64.65±8.74	-	-

611-14-3	1-Ethyl-2-methylbenzene	360	-	18.86±4.20	170.97±17.72	-	-
620-14-4	3-Eethyltoluene	800	-	21.72±0.18	150.37±2.98	0.027146	0.187958
698-87-3	Benzylmethylcarbinol	-	weak rose	0.36±0.07	-	-	-
7359-72-0	2,3,4-Trichlorotoluene	-	-	-	33.51±5.08	-	-
95-47-6	*o*-Xylene	450.23	geranium	-	17.62±1.20	-	0.039137
95-63-6	1,2,4-Trimethylbenzene	260	-	-	36.20±2.92	-	0.139217
95-73-8	2,4-Dichlorotoluene	-	-	3.01±0.85	48.82±2.66	-	-
98-82-8	Isopropylbenzene	70	-	-	42.44±4.70	-	0.606350
Subtotal				377.73	3545.15		
	Miscellaneous						
1072-83-9	2-Acethylpyrrole	58585.25	nut, cherry	1.77±0.32	5.48±0.55	0.000030	0.000087
557-31-3	Allyl ethyl ether	-	-	7.32±1.36	-	-	-
97-72-3	Isobutyric anhydride	-	-	2.05±0.86	-	-	-
17455-13-9	18-Crown-6	-	-	-	14.80±0.37	-	-
33100-27-5	15-Crown-5	-	-	-	6.91±3.01	-	-
17372-78-0	3-methylcinnoline	-	rose	-	14.02±4.28	-	-
544-01-4	Isopentyl ether	-	rose, citrus	-	5.56±0.72	-	-
Subtotal				11.14	46.77		

[1] Obtained by reviewing the literature,
[2] Flavor profile descriptions were obtained from http: //www.thegoodscentscompany.com; https: //www.chemicalbook.com,
[3] Marked "-" indicated that not detected or relevant information was not retrieved, the same as below.
